# Matrix Stiffness Drives Aggressive Phenotype in Tongue Squamous Cell Carcinoma via Mechanotransduction–Stromal Signalling

**DOI:** 10.1016/j.identj.2026.109581

**Published:** 2026-04-25

**Authors:** Watcharaphol Tiskratok, Maythwe Kyawsoewin, Rachadol Thuephut, Kansuda Ketkrathok, Chichaya Leerahakanch, Patipan Chanwises, Paiboon Jitprasertwong, Masahiro Yamada, Hiroshi Egusa, Phoonsuk Limraksasin

**Affiliations:** a Institute of Dentistry, Suranaree University of Technology, Nakhon Ratchasima, Thailand; b Centre of Excellence for Dental Implantology, Oral Health Centre, Suranaree University of Technology Hospital, Suranaree University of Technology, Nakhon Ratchasima, Thailand; c Centre of Excellence for Dental Stem Cell Biology, Faculty of Dentistry, Chulalongkorn University, Bangkok, Thailand; d Division of Mechanobiology and Biomedical-Dental Engineering, Tohoku University Graduate School of Biomedical Engineering, Sendai, Japan; e Division of Molecular and Regenerative Prosthodontics, Tohoku University Graduate School of Dentistry, Sendai, Japan; f Department of Anatomy, Faculty of Dentistry, Chulalongkorn University, Bangkok, Thailand

**Keywords:** Tongue squamous cell carcinoma, Extracellular matrix stiffness, Epithelial–mesenchymal transition, Cellular mechanotransduction, Stromal fibroblasts

## Abstract

**Objectives:**

Tongue squamous cell carcinoma (TSCC) is a highly aggressive malignancy where extracellular matrix (ECM) stiffening drives epithelial–mesenchymal transition (EMT). However, the specific mechanotransduction pathways and the distinction between primary and metastatic cell responses remain insufficiently defined. This study investigated how substrate stiffness regulates TSCC progression via a dual-regulatory mechanism: direct cell-intrinsic mechanotransduction and indirect stromal paracrine signalling.

**Methods:**

Two human TSCC cell lines with distinct origins, HSC-4 (metastatic) and HSC-7 (primary), were cultured on tunable collagen-coated polydimethylsiloxane (PDMS) substrates of varying stiffness (soft and stiff). Cell morphology, migration, proliferation, EMT marker expression, integrin and YAP expressions were assessed using wound healing assays, qRT-PCR and immunofluorescence staining. The involvement of actin cytoskeleton was examined using cytochalasin D. Additionally, the paracrine effects were evaluated by culturing TSCC cells with conditioned media from gingival fibroblasts (HGF-CM) cultured on different substrate stiffness.

**Results:**

Stiff substrates induced elongated, mesenchymal-like morphology and significantly enhanced migration in TSCC cells. Increased stiffness also upregulated EMT-associated markers (*CDH2, VIM, MMP2*), while induced YAP nuclear translocation and increased mechanosensitive integrin expression. Disruption of the actin cytoskeleton with cytochalasin D suppressed this stiffness-induced EMT marker expressions, indicating that cytoskeletal tension mediates mechanotransduction. Furthermore, HGF-CM derived from stiff substrates significantly upregulated EMT-related expression in HSC cells.

**Conclusions:**

Matrix stiffness drives TSCC progression through a dual mechanism: direct actin-mediated and YAP-associated mechanotransduction and indirect stiffness-modulated fibroblast signalling. These findings highlight that mechanical cues in the tumour microenvironment differentially regulate primary and metastatic phenotypes in TSCC.

**Clinical Significance:**

Mechanical properties of the tumour microenvironment drive TSCC progression, suggesting that ECM stiffness is likely to be associated with altered TSCC phenotypes, providing a basis for future mechanobiology-focused studies on TSCC progression and management.

## Graphical abstract

**Figure:**
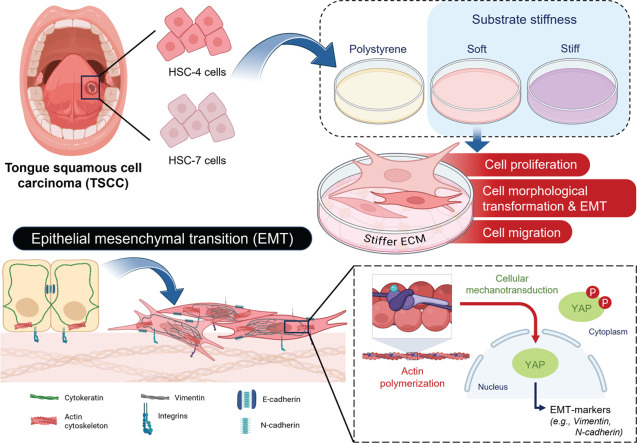

## Introduction

Oral squamous cell carcinoma (OSCC) is the predominant malignancy of the oral cavity, accounting for over 90% of oral cancers and representing a major subset of head and neck squamous cell carcinomas. Tongue squamous cell carcinoma (TSCC) is particularly aggressive, representing nearly 45% of OSCC cases and demonstrating faster progression, deeper patterns of invasion and poorer prognostic outcomes than tumours at other oral sites.^1^^,^^2^ TSCC is characterised by a high rate of cervical lymph node metastasis and local recurrence, contributing to its malignant behaviour.^3^ Despite advances in surgery and adjuvant therapies, surgical resection remains highly invasive and often associated with substantial functional and aesthetic impairment. Moreover, the 5-year survival rate for OSCC remains approximately 50%, largely due to late diagnosis, locally invasive growth and frequent regional or distant metastasis.^4^^,^^5^ These challenges highlight the importance of earlier detection and the development of less invasive, targeted treatments to enhance survival outcomes and overall patient quality of life.

OSCC pathophysiology originates from epithelial cells that acquire genetic and phenotypic changes leading to loss of polarity, basement membrane disruption and invasion into connective tissue.^6^ These changes are associated with the epithelial-to-mesenchymal transition (EMT), a dynamic process in which epithelial cells lose cell–cell adhesion such as E-cadherin and gain mesenchymal markers such as N-cadherin and vimentin.^7^^,^^8^ EMT is further characterised by cytoskeletal reorganisation and loss of cell polarity.^9^^,^^10^ Matrix metalloproteinases (MMPs), particularly MMP-2 and MMP-9, play pivotal roles in facilitating this transition, while cues from the tumour microenvironment (TME) strongly modulate EMT activation and progression.^11^^,^^12^

Mechanical remodelling of the TME, particularly extracellular matrix (ECM) stiffening, is increasingly recognised as a hallmark of cancer progression. Tumour stiffening results from excessive collagen deposition and cross-linking, disrupted matrix microarchitecture and increased interstitial fluid pressure.^7^^,^^13^ Clinically, TSCC typically manifests as firm, exophytic masses with increased keratinisation and angiogenesis.^14^ The firm texture of these lesions reflects alterations in the mechanical properties of the TME. Tumour stiffening results from excessive ECM deposition and elevated interstitial pressure due to uncontrolled tumour growth.^15^ Mechanistically, ECM stiffening promotes EMT, a cellular program that endows epithelial cancer cells with mesenchymal-like features, including increased motility and invasiveness.^16^, ^17^, ^18^ Mechanical cues can trigger EMT by activating mechanosensitive ion channels and cytoskeletal tension pathways, leading to regulate the expression of EMT-related transcription factors.^19^^,^^20^ Beyond providing structural support, the ECM actively transmits mechanical cues through integrins, focal adhesions and the actin cytoskeleton to modulate cancer cell behaviour via cellular mechanotransduction.^21^^,^^22^

However, the specific mechanosensitive behaviour of TSCC remains insufficiently defined. While previous studies have linked stiffness to dormancy or general EMT in oral cancer,^23^^,^^24^ the dual-regulatory mechanism involving both cell-intrinsic pathways and stromal crosstalk remains underexplored. Furthermore, given the heterogeneity of TSCC, it is unclear how metastatic origin influences mechanosensitivity. For instance, HSC-4 (derived from a metastatic lymph node) and HSC-7 (derived from a primary tongue tumour) may exhibit distinct adaptive responses to mechanical stress.^25^

In this study, we utilised synthetic substrates with tunable stiffness to mimic the TME of the tongue and investigate the dual-regulatory role of ECM stiffness. We specifically examined 2 distinct mechanisms:^1^ direct intrinsic mechanotransduction, to determine how stiffness regulates morphology, migration and EMT via YAP-associated and actin-mediated tension; and^2^ indirect stromal signalling, to evaluate how fibroblasts cultured on stiff substrates amplify TSCC aggressiveness via paracrine cues. We hypothesised that increasing substrate stiffness differentially modulates the behaviour of primary versus metastatic cell lines, promoting a pro-invasive phenotype through both direct mechanical cues and indirect stromal activation.

## Materials and methods

### Preparation of substrate stiffness for TSCC

A vinyl-terminated base and methyl hydrogen siloxane curing agent from commercial PDMS (Sylgard 527; Dow Corning) were prepared following a previously established method. To fabricate substrates with different stiffness levels, the vinyl-terminated base was combined with the methyl hydrogen siloxane curing agent in weight ratios of 5:4 and 1:1, producing soft (4.4 kPa) and stiff substrates (17.0 kPa), respectively. As previously reported, the stiffness values are formulation-based and literature-validated.^26^ These formulations underwent polymerisation at 65°C for 4 hours in a controlled atmosphere. For the preparation of the culture surfaces, either polystyrene culture plates or sterilised PDMS substrates were treated with oxygen plasma. The polystyrene plates were used as a standard culture reference, with primary experimental comparisons drawn between the soft and stiff PDMS substrates. All substrates were then coated with a 0.01 wt% solution of bovine dermis-derived native type I collagen solution (IAC-30; Koken Co, Ltd). The coated plates were subsequently incubated at room temperature for 90 minutes to ensure adequate adsorption of the collagen.

### TSCC culture

Two human TSCC cell lines were utilised in this study. The HSC-4 cell lines, which originate from a lymph node metastasis, were sourced from the Japanese Collection of Research Bioresources (JCRB) Cell Bank (JCRB0624; RRID:CVCL_1289) and HSC-7 cell lines (RRID:CVCL_A618) were provided by Professor Teruo Amagasa, Institute of Science Tokyo, Tokyo, Japan.^27^ Cell line authentication was based on source verification from the cell bank and was further confirmed in our laboratory through routine monitoring of morphological characteristics and growth kinetics. Prior to all experiments, the mycoplasma testing method was verified using DNA staining with DAPI (4′,6-diamidino-2-phenylindole) to detect mycoplasmas under fluorescence microscope.^28^ Both HSC-4 (passage No. 4-14) and HSC-7 cells (passage No. 4-11) were cultured in high-glucose Dulbecco’s Modified Eagle Medium (DMEM) (Gibco, Grand Island, NY, USA), supplemented with 10% fetal bovine serum (Gibco), 2 mM L-glutamine (Gibco), 100 U/mL penicillin (Gibco), 100 µg/mL streptomycin (Gibco) and 5 µg/mL amphotericin B (Gibco). The cells were incubated at 37°C in a humidified atmosphere with 5% CO_2_, with the culture media being refreshed every 48 hours. Once the cells reached approximately 80% confluence, they were subcultured at a ratio of 1:3 using 0.25% trypsin-EDTA (Gibco).

### Human gingival fibroblasts (HGFs) cultures

HGFs were extracted from 5 periodontally healthy molars belonging to individuals aged 18 to 25 years, who visited the Oral Health Centre at Suranaree University of Technology Hospital. The third molar tooth extractions were carried out either due to impaction or for orthodontic considerations. The isolation and cultivation of the HGFs adhered to established protocols from previous research.^29^ This study received approval from the Human Research Ethics Committee of Suranaree University of Technology (EC-68-0078).

The HGFs were cultured in a growth medium formulated with high-glucose Dulbecco's Modified Eagle Medium (DMEM) (Gibco, Grand Island), supplemented with 10% fetal bovine serum (Gibco), 2 mM L-glutamine (Gibco), 100 U/mL penicillin (Gibco), 100 µg/mL streptomycin (Gibco) and 5 µg/mL amphotericin B (Gibco). The cultures were maintained at 37°C in a 5% CO2 environment. Once the cells reached 80% confluence, they were detached using a solution of 0.25% trypsin and 1 mM ethylenediaminetetraacetic acid (Gibco) and subsequently reseeded in HGF growth medium on collagen-coated substrates within 24-well culture-grade polystyrene plates or on soft or stiff PDMS materials. The growth medium was refreshed every 48 hours and passages 3 to 7 were employed for all experiments conducted in this study.

Condition media from HGF cultures on each substrate was collected after 24 hours. HGF-CM in each was centrifuged at 3000 g for 15 minutes to remove cellular debris and stored at –80°C in preparation for subsequent co-culture experiments.

### HSC cell lines co-culture with condition medium of HGFs

The HSC-4 and HSC-7 cells were co-cultured with a 1:5 ratio of supernatant taken from HGFs on polystyrene, as well as on soft and stiff substrates, with fresh growth medium for 3 days respectively and the FBS was adjusted so that all groups were exposed to a standardised final FBS of 10%. This setup was employed to conduct a scratch migration assay and to assess gene and protein expression.

### Cell proliferation assay

HSC-4 cells were seeded at a density of 1 × 10⁵ cells per well in a 12-well culture plate on oxygen plasma and collagen-coated PDMS with varying stiffness levels, with polystyrene as the control. The cells were incubated for 24 and 72 hours at 37°C in an atmosphere containing 5% CO₂. Following incubation at the specified time points, the cells were treated with MTT (3-(4,5-dimethyl-2-thiazolyl)-2,5-diphenyl-2H-tetrazolium bromide) solution (Invitrogen) for 30 minutes at 37°C to facilitate the formation of formazan crystals. These crystals were subsequently dissolved in a solution of dimethyl sulfoxide and glycine. Viable cells converted the yellow MTT solution into purple formazan crystals. The absorbance of the resulting product was measured using a microplate reader (BioTek Synergy H1 Hybrid Reader; BioTek Instruments Inc) at a wavelength of 570 nm to evaluate cell proliferation.

### Cell morphometry analysis

The cells were examined using a phase microscope. with a phase contrast microscope. Using ImageJ software (National Institutes of Health), we conducted an analysis of cell morphometry, focusing on parameters such as cell area, perimeter and circularity derived from the phase images.

### Scratch wound healing migration assay

The cell migration of HSC-4 cells was assessed using a scratch wound healing migration assay. The cells at a density of 1 × 10^5^/well were seeded into 12-well plates, then maintained in growth media with the humidified incubator overnight. A vertical scratch was created by a 1000-μL pipet tip, after which the detached cells and debris were removed by washing with phosphate-buffered saline (PBS). The cells were incubated in growth medium with serum-free. Wound closure was monitored and images were captured at 0, 24 and 48 hours using an inverted microscope (Nikon TS2). The extent of the scratch wound was analysed with ImageJ software and the percentage of wound closure was calculated using a specific formula.

The percentage  (%) of wound closure = [(T_0_ - T_N_)/T_0_] × 100

T_0_ = Initial wound area (0 hour)

T_N_ = Remaining wound area at 24 hours or 48 hours

### Inhibitor treatment

To investigate the mechanism of mechanotransduction, HSC-4 were cultured PDMS surfaces that were coated with oxygen plasma and collagen, varying the stiffness of the substrates. The cells were seeded at a density of 1 × 10⁵ cells per well in a 12-well culture plate, with polystyrene serving as the control material. The cells were then incubated at 37°C with 5% CO₂ for 3 days. Treatment with 5 μM cytochalasin D^30^ (Sigma-Aldrich, St. Louis), a strong inhibitor of actin polymerisation, was applied to all experimental groups, excluding the polystyrene control, 3 hours prior to sample collection.

### Reverse transcription–polymerase chain reaction (RT–PCR)

RNA isolation was done using RiboEx total RNA isolation solution (GeneAll). Subsequently, 1 μg RNA was converted to complementary DNA (cDNA) employing the ImProm-II Reverse Transcription System (Promega), followed by real-time PCR analysis. The PCR cycling conditions comprised an initial denaturation at 95°C for 30 seconds, followed by 40 amplification cycles of 95°C for 3 seconds and 60°C for 30 seconds. The real-time RT-PCR was performed using the Green Master Mix (Promega) on a Roche real-time PCR system, adhering strictly to the manufacturer's protocols. For quantifying target gene expression levels, the comparative cycle time (ΔΔCT) method was utilised, with glyceraldehyde 3-phosphate dehydrogenase (*GAPDH*) serving as the reference housekeeping gene. Detailed sequences of the primers used for real-time RT-PCR are provided in Supplementary Table 1.

### Immunofluorescence staining and analyses

HSC-4 cells grown on collagen-coated polystyrene culture plates and PDMS were fixed using a 4% paraformaldehyde phosphate buffer solution (Thermo Fisher Scientific) for a duration of 15 minutes. After fixation, the cells were rinsed with PBS and subsequently treated with a blocking buffer to inhibit nonspecific protein binding. This blocking buffer consisted of 3% bovine serum albumin (BSA) (Sigma-Aldrich), 0.1% Triton-X (USBiological Life Sciences) and 0.01% Tween 20 (Sigma-Aldrich) for 1 hour. To identify specific cellular markers, the cells underwent a series of processes including fixation, permeabilisation and blocking of nonspecific proteins. They were then incubated overnight at 4°C with either an anti-vimentin monoclonal antibody (V9, sc-6260: diluted 1:200 in 3% BSA, Santa Cruz Biotechnology), anti-N cadherin monoclonal antibody (ab98952, diluted 1:200 in 3% BSA) or an anti-YAP monoclonal antibody (sc-101199, diluted 1:200 in 3% BSA, Santa Cruz Biotechnology). Following this step, the samples were treated with 1/1000 Alexa Fluor 488-conjugated goat antimouse IgG (Thermo Fisher Scientific) for 1 hour at room temperature. Additionally, the samples were stained with 1/1000 Rhodamine-phalloidin (Cytoskeleton Inc), using PBS as the diluent. After thorough PBS washing, the samples were mounted on slides with an antifade mounting medium containing DAPI (VectorLab). Immunofluorescent microscopy and analyses were performed using a fluorescence microscope (CX43; Olympus).

### Statistical analysis

Statistical analyses were conducted using GraphPad Prism 9 software. We evaluated the normality of the data employing the Shapiro–Wilk test. For experiments analysing a single independent variable across multiple groups, a parametric one-way ANOVA was utilised, followed by Tukey's multiple comparison test. For time-course experiments involving 2 independent variables, a two-way ANOVA was utilised, followed by Tukey's multiple comparison tests. In cases where data exhibited unequal variances, Welch’s test was applied. A *P*-value of less than .05 was considered statistically significant.

## Results

### Substrate stiffness regulates cell viability and morphology of TSCC

**Fig. 1:**
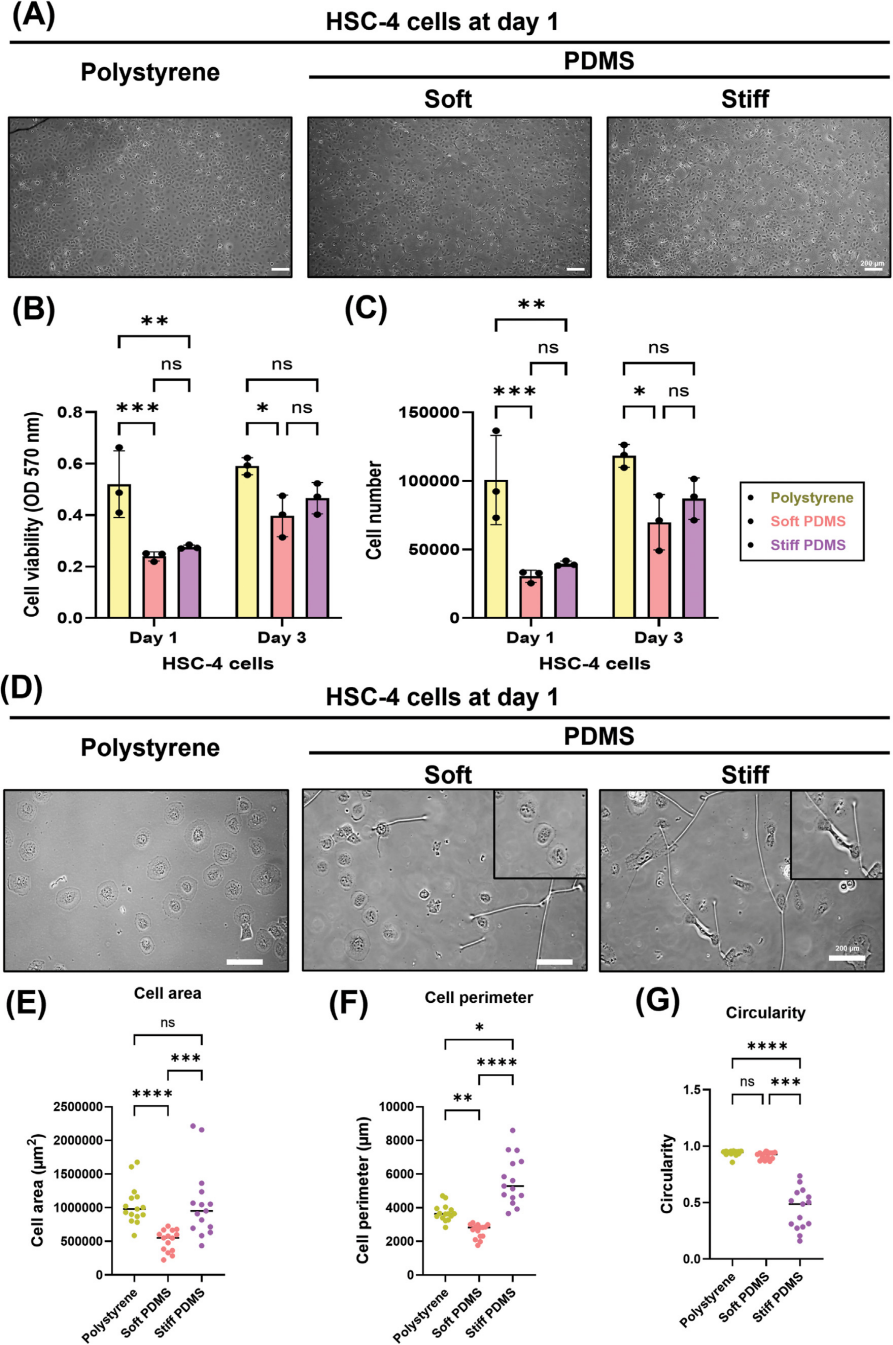
Substrate stiffness regulates the cell viability and morphology of HSC-4 cells. (A) representative images of cell morphology (B) quantitative analysis of cell viability (C) cell number (D) representative images of cell shape (E) quantitative analysis of cell area (F) cell perimeter (G) circularity of HSC-4 cells on various substrate stiffness conditions are determined at day 1 and day 3. Data were statistically analysed by two-way ANOVA followed by Tukey’s multiple comparison tests (B-C, n = 3) and Kruskal–Wallis tests followed by multiple comparison tests (E-G, n = 15: * P < .05, ** P < .01, *** P < .001, **** P < .0001; ns, no significant difference). Data are presented as the mean ± standard deviation (SD), with different letters indicating statistically significant differences between multiple groups. OD, optical density; PDMS, polydimethylsiloxane.

To investigate the effects of substrate stiffness on cell proliferation and morphology of HSC-4 cells, cells were cultured on oxygen plasma– and collagen-coated PDMS substrates with soft and stiff substrates. The MTT assay demonstrated that the relative proliferation of HSC-4 cells was significantly reduced on both soft (*P* < .001) and stiff (*P* < .01) substrates on day 1. By day 3, a significant decrease in proliferation was observed only in cells cultured on the soft PDMS substrate when compared with the polystyrene control (Figure 1A-C).

Morphological evaluation after 1 day of culture revealed distinct differences in cell shape across the substrate stiffness conditions (Figure 1D). Cells grown on the control and stiff substrates exhibited greater spreading areas, whereas those on the soft substrate showed a smaller cell area (Figure 1E). Furthermore, the cell perimeter was significantly higher on the stiff substrate compared with the other groups (Figure 1F). In contrast, cell circularity was markedly reduced under stiff substrate conditions relative to both the soft and control surfaces (Figure 1G). These findings indicated that HSC-4 cells cultured on stiff substrates acquire an elongated, mesenchymal-like morphology, whereas those on soft and polystyrene substrates retain a more polygonal appearance.

### Substrate stiffness induces cell migration of HSC-4 cells

**Fig. 2:**
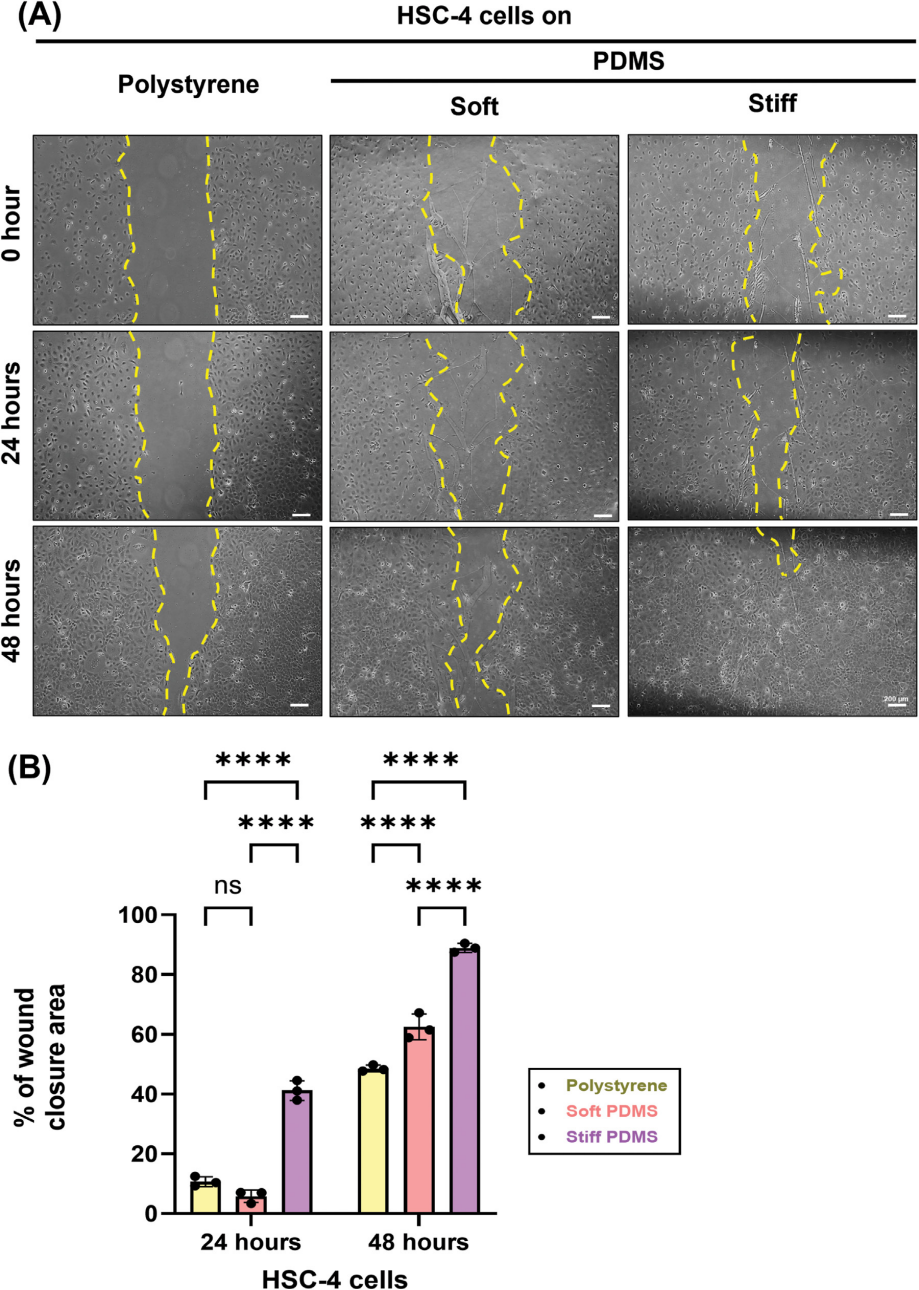
Substrate stiffness impacts the cell migration of HSC-4 cells. (A) Representative images of scratch wound assay of HSC-4 under various stiffness conditions at 0, 24 and 48 hours (B) Quantitative analysis of percentage of wound closure under various stiffness conditions at 24 and 48 hours. Data were statistically analysed by two-way ANOVA followed by Tukey’s multiple comparison tests (n = 3: * P < .05, ** P < .01, *** P < .001, **** P < .0001, ns, no significant difference). Data are presented as the mean ± standard deviation (SD). PDMS, polydimethylsiloxane.

To investigate the effect of substrate stiffness on the migratory behaviour of HSC-4 cells, migration wound healing assays were performed under different stiffness conditions. The result demonstrated that HSC-4 cells on stiff substrate showed a marked increase in percentage of wound closure area at both 24-hour and 48-hour intervals compared to soft stiffness and polystyrene control (Figure 2A-B). Our finding suggested that ECM stiffness-controlled the cell migration of TSCC cell line.

### Substrate stiffness modulates EMT-marker expression in TSCC cell lines

**Fig. 3:**
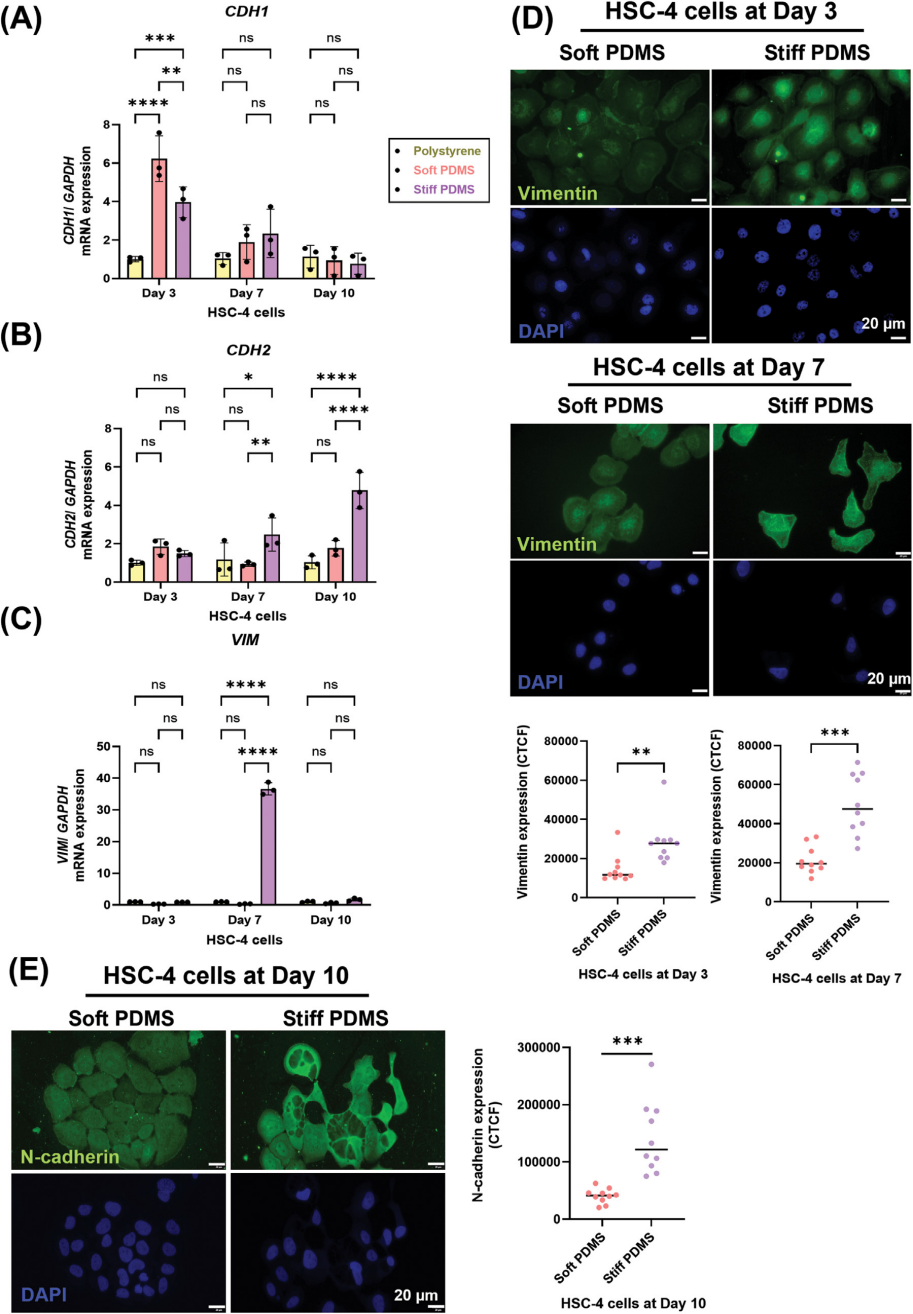
Substrate stiffness modulates EMT-related expression in HSC-4 cells. HSC-4 cells were incubated under various stiffness conditions for 3, 7 and 10 days. Real-time qRT-PCR was performed to detect mRNA expression levels of (A) *CDH1* (B) *CDH2* and (C) *VIM*. The expression of *GAPDH* was used as an internal control. (D) Immunofluorescence analysis was performed to detect the vimentin protein expression (green). The nuclei (blue) were stained using DAPI, respectively. (E) Immunofluorescence analysis was performed to detect the N-cadherin protein expression (green). The nuclei (blue) were stained using DAPI, respectively. Scale bars: 20 μm. Data were statistically analysed by two-way ANOVA followed by Tukey’s multiple comparison tests (A-C: n = 3) and Welch’s test (D-E: n = 10, * P < .05, ** P < .01, *** P < .001, **** P < .0001, ns, no significant difference). Data are presented as the mean ± standard deviation (SD). *CDH1*, E-cadherin; *CDH2*, N-cadherin; PDMS, polydimethylsiloxane; *VIM*, Vimentin, glyceraldehyde-3-phosphate dehydrogenase (GAPDH).

To evaluate the effect of substrate stiffness on EMT-related expression in HSC-4 cells, mRNA levels of *CDH1* (E-cadherin), *CDH2* (N-cadherin), *VIM* (Vimentin), *MMP2, MMP9* and *Ki67* were determined by qRT-PCR after 3, 7 and 10 days of incubation. The results showed that *CDH1* expression was decreased on day 3 under stiff substrate conditions compared with soft substrate in HSC-4 cells (Figure 3A). In contrast, *CDH2* level was significantly elevated on stiff substrates at day 7 and 10 (Figure 3B). Furthermore, vimentin expression was significantly increased in HSC-4 cells cultured on the stiff substrate at day 7 (Figure 3C). These results further supported by immunofluorescence staining that confirmed a corresponding increase at the vimentin protein level (Figure 3D) and N-cadherin protein level (Figure 3E), indicating that increased rigidity promoted EMT expression. In addition, *MMP2* mRNA expression was significantly higher in cells on the stiff substrate compared with those on the soft substrate on day 3, although no difference was observed by day 7. On the contrary, *MMP9* and *Ki67* expression showed no significant differences between soft and stiff substrate conditions at either time point (Supplementary Figure S1A-C)

**Fig. 4:**
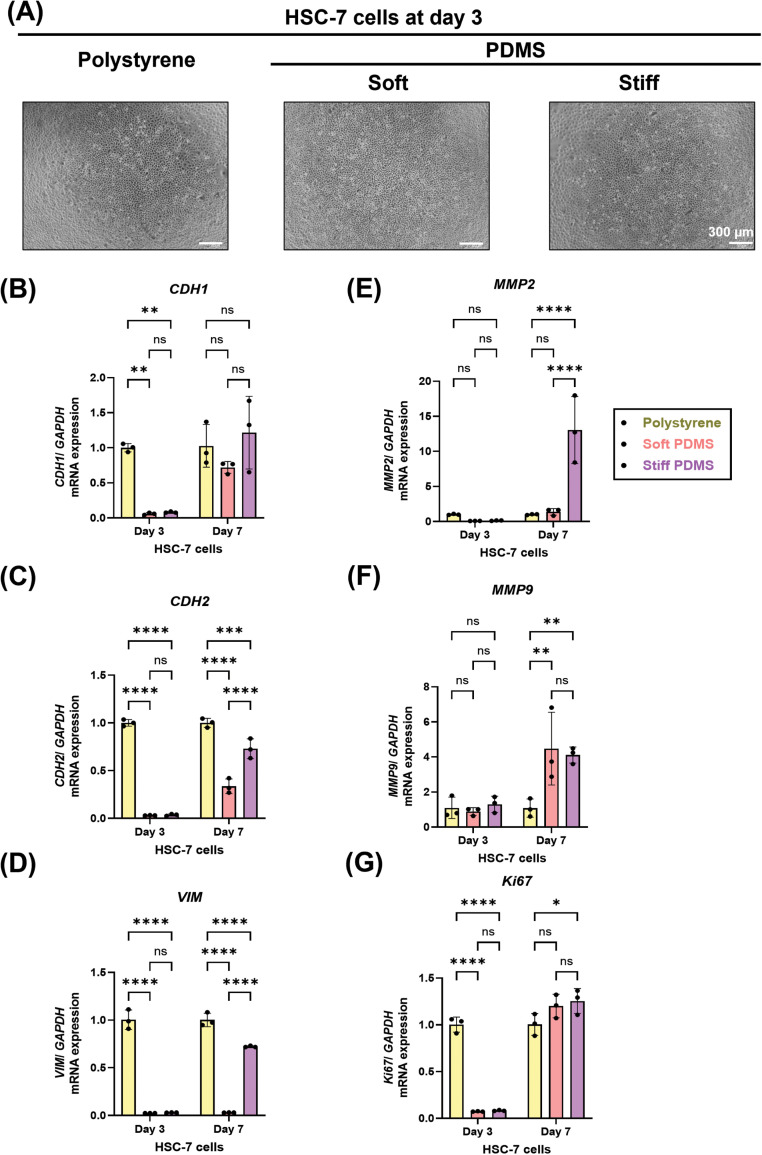
Substrate stiffness modulates EMT-related expression by HSC-7 cells. HSC-7 cells were incubated under various stiffness conditions for 3 and 7 days. (A) Representative images of morphology of HSC-7 cells at day 3. Real-time RT-PCR was performed to detect mRNA expression levels of (B) *CDH1* (C) *CDH2,* (D) *vimentin,* (E) *MMP2,* (F) *MMP9 and* (G) *Ki 67.* The expression of *GAPDH* was used as an internal control. Data were statistically analysed by two-way ANOVA followed by Tukey’s multiple comparison tests (n = 3: * P < .05, ** P < .01, *** P < .001, **** P < .0001, ns, no significant difference). Data are presented as the mean ± standard deviation (SD). *CDH1*, E-cadherin; *CDH2*, N-cadherin; *GAPDH*, glyceraldehyde-3-phosphate dehydrogenase; *MMP9*, Matrix metalloproteinase-9; PDMS, polydimethylsiloxane; *VIM*, Vimentin; *MMP2*, Matrix metalloproteinase-2.

To determine the influence of substrate stiffness on EMT-related gene expression in HSC-7 cells, the cells were cultured on substrates with different stiffness levels (Figure 4A). While EMT marker expression showed no significant differences between conditions on day 3, stiff substrates significantly upregulated mRNA expression of *CDH2, VIM* and *MMP2* on day 7 (Figure 4C-E). In contrast, *CDH1* and *MMP9* expressions remained unchanged between soft and stiff PDMS at both time points (Figure 4F and G). These findings illustrate the complex, cell line–specific effects of mechanical stiffness on EMT marker regulation in TSCC.

### Mechanotransduction mechanisms involved in the influence of substrate stiffness in TSCC

**Fig. 5:**
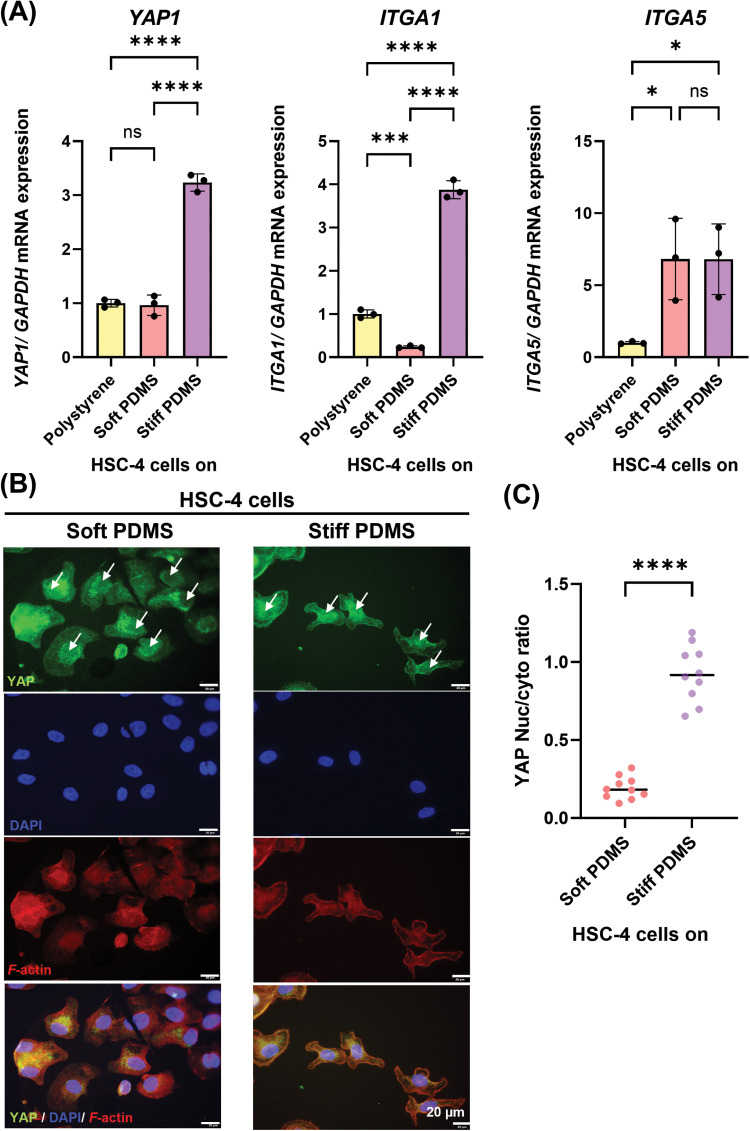
Mechanotransduction mechanism involved in the influence of substrate stiffness in HSC-4 cells. Real-time RT-PCR was performed to detect mRNA expression levels of (A) *YAP, ITGA1* and *ITGA5.* The expression of *GAPDH* was used as an internal control. (B) Immunofluorescence analysis was performed to detect the protein expression and localisation of YAP (green). The cytoskeleton (F-actin; red) and nuclei (blue) were stained using rhodamine-phalloidin and DAPI, respectively. (C) A corresponding histogram of Nuc/cyto YAP ratio from immunofluorescence staining. YAP prio Scale bars: 20 μm. Data were statistically analysed by one-way ANOVA followed by Tukey’s multiple comparison tests (A: n = 3) and Welch’s test (C: n = 10 * P < .05, ** P < .01, *** P < .001, **** P < .0001, ns, no significant difference). Data are presented as the mean ± standard deviation (SD). GAPDH, Glyceraldehyde-3-phosphate dehydrogenase; ITGA1, Integrin alpha-1; ITGA5, Integrin alpha-5; PDMS, polydimethylsiloxane; YAP, Yes-associated protein.

To investigate whether matrix stiffness regulates mechanotransduction mechanism in TSCC cell lines, we evaluated the expression of mechanosensory integrins, including *ITGA1* and *ITGA5,* as well as the mechanotransduction-associated transcriptional coactivator YAP. The results demonstrated that HSC-4 cells cultured on the stiff substrate exhibited significantly increased mRNA expression of *ITGA1* and *YAP1*, whereas *ITGA5* expression did not differ between the soft and stiff substrates (Figure 5A). Furthermore, nuclear translocation of YAP protein was observed in HSC-4 cells on stiff substrate, while cells on the soft PDMS showed weak nuclear YAP signals and predominantly cytoplasmic YAP localisation on day 3 (Figure 5B). This result further supported by YAP nuclear/cytoplasm ratio (Figure 5C). Similarly, stiff substrates significantly increased *YAP1* mRNA expression in HSC-7 cells compared with soft substrates (Supplementary Figure S2A). To further evaluate downstream YAP signalling activity, we evaluated the expression of the canonical YAP target gene *CTGF*, which was significantly upregulated in cells cultured on the stiff substrate (Supplementary Figures S1D and S2B). These findings suggest that TSCC cellular responses to matrix stiffness are closely associated with mechanotransduction pathways.

### Substrate stiffness regulates EMT marker expression in TSCC through mechanotransduction mechanism

**Fig. 6:**
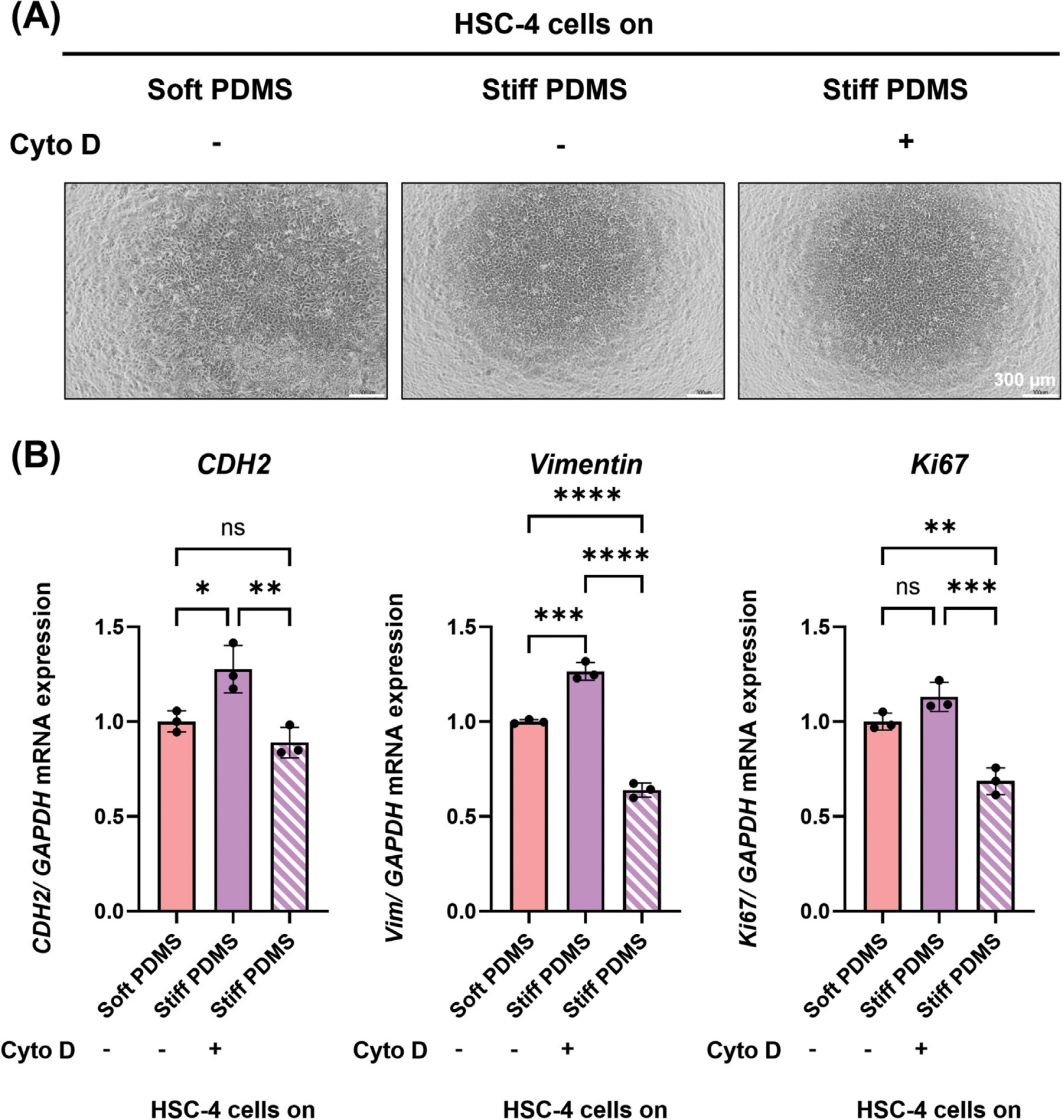
The impact of substrate stiffness on EMT markers expression by OSCC through mechanotransduction mechanism. (A) Representative images of HSC-4 cells which were incubated under stiffness conditions for 3 days with or without-3-hour cytochalasin treatment. (B) Real-time RT-PCR was performed to detect mRNA expression levels of (A) *CDH2, VIM* and *Ki67.* The expression of *GAPDH* was used as an internal control. Data were statistically analysed by one-way ANOVA followed by Tukey’s multiple comparison tests (n = 3: * P < .05, ** P < .01, *** P < .001, **** P < .0001, ns, no significant difference). Data are presented as the mean ± standard deviation (SD). *CDH2*, N-cadherin; PDMS, polydimethylsiloxane; *VIM*, Vimentin.

To further confirm substrate stiffness controls EMT-related gene expression in HSC-4 cells through cellular mechanotransduction, cells were treated with and without cytochalasin D, an inhibitor of actin polymerisation, after 3 days of culture under stiff substrate conditions. After inhibitor treatment, the cells remained adherent to the substrates (Figure 6A). Notably, cytochalasin D effectively suppressed the stiffness-induced upregulation of EMT markers, including *CDH2, VIM* and *Ki67* (Figure 6B). These findings demonstrated that cytoskeletal-related actin polymerisation is involved in transmitting mechanical cues that regulate EMT in TSCC.

### Conditioned media of HGFs (HGF-CM) influences the behaviour of HSC cells under different substrate stiffness

**Fig. 7:**
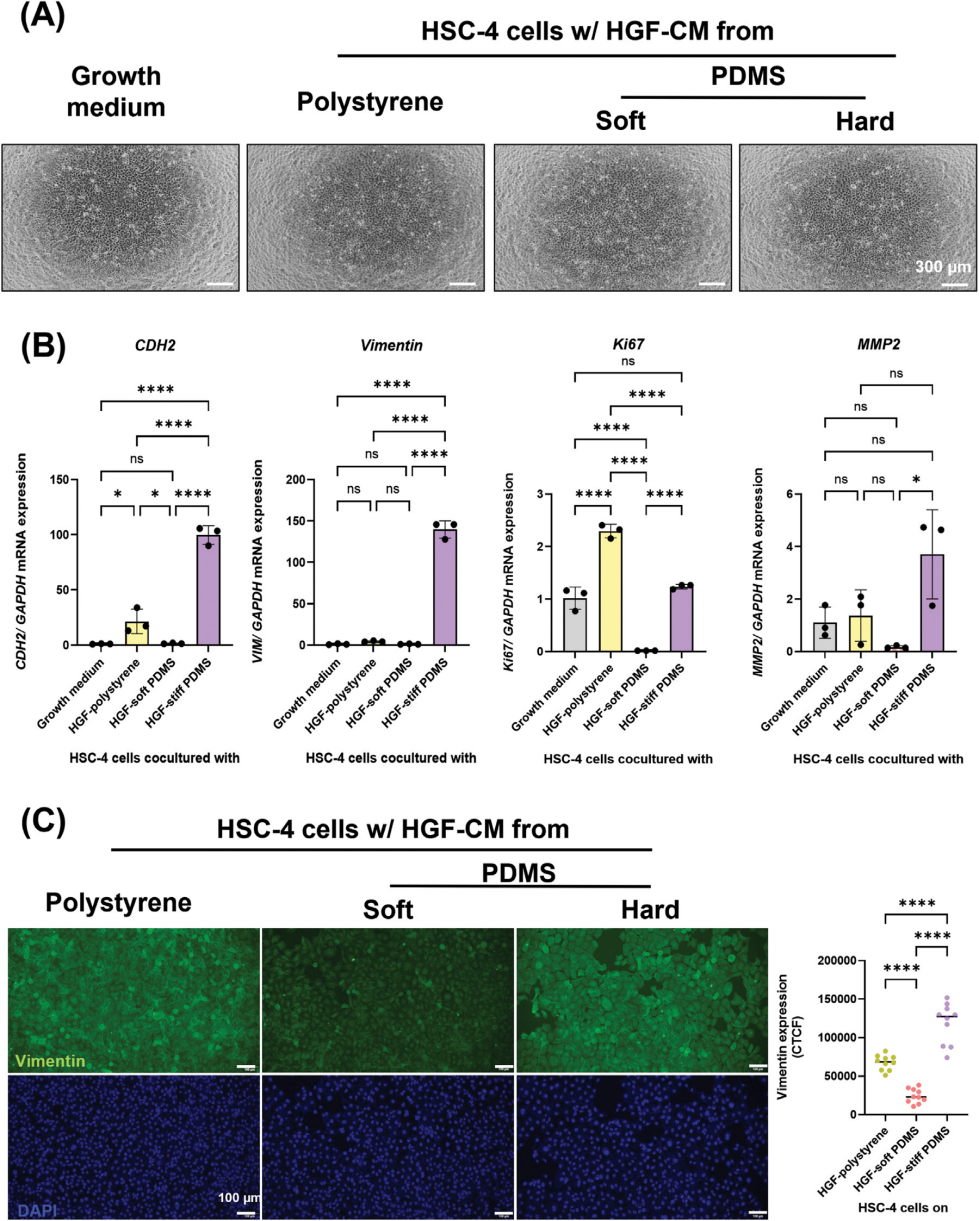
The effect of conditioned media of human gingival fibroblasts (HGF-CM) on the behaviour of HSC-4 under different substrate stiffness. (A) Representative images of HSC-4 cocultured with HGF-CM for 3 days, Scale bars: 300 μm. Real-time RT-PCR was performed to detect mRNA expression levels of (B) *CDH2, VIM, Ki67* and *MMP2.* The expression of *GAPDH* was used as an internal control. (C) Immunofluorescence analysis was performed to detect the vimentin protein expression (green). The nuclei (blue) were stained using DAPI, respectively. Data were statistically analysed by one-way ANOVA followed by Tukey’s multiple comparison tests (n = 3) and Welch’s test (C: n = 10 *P < .05, ** P < .01, *** P < .001, **** P < .0001, ns, no significant difference). Data are presented as the mean ± standard deviation (SD). *CDH2*; N-cadherin, *GAPDH*, glyceraldehyde-3-phosphate dehydrogenase; *MMP2*, Matrix metalloproteinase-2; PDMS, polydimethylsiloxane; *VIM*, Vimentin.

HSC-4 cells cocultured with HGF-CM did not reveal any significant alterations in cell morphology and cell number compared with the control group (Figure 7A). However, HSC-4 cells treated with HGF-CM derived from HGFs cultured on the stiff substrates exhibited a significant increase in *CDH2, VIM, Ki67* and *MMP2* mRNA expression on day 3 (Figure 7B). This transcriptional pattern was supported by immunofluorescence staining, which demonstrated stronger vimentin protein signals in HSC-4 cells cocultured with HGF-CM from the stiff substrate compared with those treated with HGF-CM from the soft substrate (Figure 7C). In addition, HSC-7 cells cocultured with HGF-CM derived from HGFs cultured on the stiff substrates slightly increased the gene expression of *CDH2, VIM* and *Ki67* compared with those treated with HGF-CM from the soft substrate (Supplementary Figure S3).These findings indicated that HGF-CM can modulate HSC cell behaviour in a stiffness-associated manner, suggesting a potential role of fibroblast-derived paracrine signalling in TSCC mechanobiology.

## Discussion

Our study demonstrates a prominent influence of ECM stiffness on the behaviour of TSCC cell lines. Increased substrate stiffness enhanced cell migration and induced an elongated morphology, consistent with a shift toward a mesenchymal-like phenotype. Stiff substrate also upregulated EMT-related gene expression and promoted nuclear localisation of YAP, which is associated with cellular mechanotransduction. The paracrine experiments further demonstrated that HSC cell lines cocultured with conditioned media from HGFs cultured on stiff substrates exhibited increased expression of EMT markers. Together, these results suggest that the mechanical microenvironment regulates TSCC behaviour both directly, through cancer cell mechanosensing and indirectly, through stiffness-dependent stromal fibroblast signalling. This dual regulation is associated with pro-migratory and invasive behaviour, highlighting ECM stiffness as a critical mechanical cue driving TSCC progression.

In this study, we utilised substrate stiffness values of 4.4 kPa and 17.0 kPa to model physiologically relevant mechanical conditions of the oral tumour microenvironment. The lower stiffness represents a relatively compliant microenvironment comparable to softer regions of oral tissues, while the higher stiffness reflects the mechanical stiffening observed during tissue remodelling and tumour progression. Previous studies have reported that oral squamous cell carcinoma tissues exhibit considerable mechanical heterogeneity, premalignant lesions such as squamous cell hyperplasia can exhibit stiffness values of approximately 13 to 21 kPa.^31^ Moreover, the stiffer regions of dysplastic oral tumours have been reported to reach values approaching 20 kPa.^23^

Matrix stiffness is a hallmark of the TME that impacts cancer progression. Increased ECM stiffness triggers biomechanical signals that regulate cellular behaviour^15^^,^^32^ and promotes the proliferation, progression and survival of cancer cells through integrin-mediated adhesion and signalling pathways.^33^ Previous studies have shown that the mechanical properties of ECM induced the abnormal activation of pathways such as the ERK1/2-YAP, FAK/Src signalling cascades, which are crucial for cytoskeletal rearrangement and cell motility.^34^^,^^35^ The relationship between ECM stiffness and cellular response is significant in the context of TSCC, as invading tumour cells typically display high adaptability to the mechanical limitations of their microenvironment.^16^, ^17^, ^18^ Consequently, the physical and mechanical characteristics of tumour stroma may contribute to the aggressiveness and invasiveness of TSCC.^22^

Stiff PDMS substrates significantly enhances the cell migration of HSC-4 cells, supporting previous study that ECM mechanics influence cancer cell behaviour, including EMT-related expression, invasion and metastasis,^36^ such as the promotion of hepatocellular carcinoma migration via MAPK–YAP signalling on stiffer substrate.^37^ Importantly, this enhanced wound closure reflects active cellular migration rather than a proliferation bias. The assay was conducted under serum-free conditions to restrict cell division and our MTT data confirmed comparable baseline proliferation rates across all substrates (Figures 1A-C). The increased cell migration of HSC-4 cells on rigid substrates is closely associated with the mechanical cues that characterise TSCC aggressiveness.

TSCC cells exhibited notable morphological changes when cultured on varying stiffness, particularly cells on stiff substrate adopted an elongated, mesenchymal-like shape, which is associated with enhanced epithelial-mesenchymal transition (EMT). This stiffness-dependent shift in cell shape aligns with previous findings showing that increased mechanical rigidity modulates the morphology of human glioblastoma cells^38^ and oral cancer cells.^23^ Similarly, elevated matrix stiffness creates a dormant subpopulation with increased EMT in oral cancer cells.^24^ The alterations in cell morphology imply a potential mechanism by which substrate properties may affect tumour behaviour and progression.

The stiffness of PDMS substrates influences EMT-related gene and protein expression in TSCC cells. Increased matrix stiffness has been shown to elevate vimentin expression and reduce *CDH1* levels in pancreatic cancer cells,^39^ indicating a shift towards a more mesenchymal phenotype. Similarly, stiffer matrix stiffness upregulated *MMP2* and *MMP9* expression in hepatocellular carcinoma cells through PI3K/Akt signalling pathway,^40^ thereby enhancing cancer invasiveness through increased migration potential of cancer cells. These findings point out the importance of the mechanical properties of the cellular environment which influences the behaviour of the cancer cells.

Our results demonstrated that substrate stiffness differentially regulates EMT-related gene expression and phenotypic behaviour in HSC-4 and HSC-7 cells, reflecting their distinct anatomical origins and baseline mechanosensitive profiles.^41^ HSC-7 cells, which are derived from a primary tumour, displayed a more immediate and sustained mesenchymal profile on stiff substrates, with upregulation of *CDH2, VIM* and *MMP2* on day 7. This suggests that primary-derived cells actively preparing for local invasion are inherently primed to respond rapidly to microenvironmental stiffening. Interestingly, *CDH1* expression in HSC-7 remained stable regardless of stiffness, suggesting these cells may be less dependent on the mechanoregulation of epithelial markers. In contrast, HSC-4 cells are derived from a lymph node metastasis. On stiff substrates, HSC-4 cells exhibited an early downregulation of E-cadherin gene expression at Day 3, suggesting an initial mechanosensitive shift away from epithelial integrity. Indeed, with prolonged exposure, stiff substrates promoted an EMT shift in HSC-4 cells, characterised by increased expression of N-cadherin, vimentin and an early elevation of *MMP2*. The consistent upregulation of vimentin at day 7 further supports a stiffness-driven enhancement of mesenchymal traits. Notably, *MMP9* and *Ki67* remained unchanged, indicating that mechanical cues selectively modulate EMT markers and matrix remodelling enzymes without broadly influencing proliferation. These findings highlight that the source of the TSCC cell line significantly shapes its mechanotransduction dynamics with primary versus metastatic origins^42^ dictating how cancer cells phenotypically adapt to ECM rigidity.

YAP, a key transcriptional coactivator, was enhanced as matrix stiffness increased, consistent with lung adenocarcinoma studies.^43^ YAP function facilitates the integration of upstream mechanical signals to drive cancer progression.^44^^,^^45^ These observations coincide with our result, which revealed that HSC-4 cells on stiff substrates exhibited increased YAP nuclear localisation. Furthermore, integrins play a significant role as mechanosensor in initiation, progression and metastasis of tumors.^46^ Specifically, integrin alpha-5 has been shown to promote the proliferation, migration and invasion of OSCC.^47^ Our findings revealed the co-localisation of YAP with upregulation of integrin alpha-1, indicating that ECM stiffness is associated with the aggressive phenotypes of TSCC through integrin-mediated mechanosensing.

In addition, cytochalasin D treatment attenuated the expression of key EMT markers in HSC-4 cells on stiff substrate by inhibition of actin polymerisation.^48^ Matrix stiffness is known to regulate cytoskeletal organisation through mechanosensitive proteins such as vinculin.^49^ Furthermore, matrix stiffness has been shown to modulate EMT marker expression in human adenocarcinoma cells.^50^ In the present study, cytochalasin D was primarily utilised to demonstrate that cytoskeletal integrity and actin tension are related to the stiffness-induced EMT changes and YAP-associated pathway observed in HSC-4 cells. Accordingly, the suppression of stiffness-induced EMT markers following cytochalasin D treatment, suggesting that actin cytoskeletal tension is associated with cancer cell responses to mechanical cues. However, cytochalasin D affected broad effects on actin dynamics preclude establishing a YAP-specific causality. Therefore, future investigations employing direct YAP perturbation, such as siRNA-mediated knockdown or specific pharmacological inhibitors,^51^ are necessary to isolate the direct role of YAP in mediating these phenotypes.

Cancer-associated fibroblasts (CAFs) are a dominant stromal component of the tumour microenvironment (TME) and critical mediators of OSCC progression. CAFs arise from resident stromal cells, including gingival fibroblasts, which undergo activation and phenotypic reprogramming in response to tumour-derived factors. Upon activation, CAFs express the expression of mesenchymal markers such as alpha-smooth muscle actin and vimentin and secrete ECM components and cytokines that remodel the TME and promote tumour growth, invasion and metastasis.^52^ Previous study demonstrated that conditioned media from human gingival fibroblasts (HGF-CM) can serve as a functional in vitro model of CAF-mediated paracrine signaling.^53^ In the present study, HSC cell lines treated with HGF-CM derived from fibroblasts cultured on stiff substrates exhibited increased expression of EMT-related markers. In our conditioned media experiments, we utilised primary human gingival fibroblasts (HGFs) as a representative model for the oral cancer-stroma crosstalk.^53^^,^^54^ Importantly, factors secreted by HGFs on stiff substrates significantly enhanced TSCC progression. This aligns with previous study that matrix rigidity acts as a potent mechanical cue, activating normal fibroblasts into a CAF-like phenotype. For example, CAFs are known to drive EMT and invasion in neighbouring carcinoma cells through the secretion of factors, such as TGF-β1 and CXCL12.^55^ Furthermore, in OSCC specifically, activated oral fibroblasts upregulate inflammatory pathways and secrete pro-tumorigenic cytokines such as IL-8 and CXCL5.^54^ We hypothesised that the stiff substrates in our study mechanically induced a similarly pro-migratory secretome in the HGFs. While the current findings clearly establish the mechanical dependency of this paracrine signalling, identifying the specific soluble factors within this stiffness-regulated secretome remains an important direction for future research.

In summary, this study provides strong evidence that ECM stiffness significantly alters the migratory and morphological characteristics of TSCC, implicating EMT-related markers in the process. The findings advocate for further exploration into the mechanobiological factors that drive TSCC progression. Understanding these relationships may improve the management of TSCC by addressing the mechanical conditions affecting tumour cells. Future research is needed to explore the application of ECM stiffness modulation in animal model and clinical translation for TSCC. Integrating these mechanobiological considerations into oral cancer research and clinical practice may ultimately facilitate more effective, patient-specific approaches to TSCC management and improve clinical outcomes.

## Conclusions

Extracellular matrix stiffness (ECM) critically regulates *in vitro* tongue squamous cell carcinoma (TSCC) progression by directly promoting mesenchymal-like morphological changes, enhanced migration and epithelial–mesenchymal transition through actin-mediated and YAP-associated mechanotransduction. Furthermore, matrix stiffness indirectly regulates tumour aggressiveness through modulated fibroblast paracrine signalling. Together, these findings highlight the mechanical tumour microenvironment in shaping TSCC phenotypes, providing a basis for future mechanobiology-focused studies on TSCC progression

## Author contributions

**Watcharaphol Tiskratok**: Conceptualisation, Methodology, Investigation, Data curation, Formal analysis, Writing – original draft, Writing – review and editing, Project administration, Funding acquisition, Supervision. **Maythwe Kyawsoewin:** Methodology, Investigation, Data curation, Writing – original draft, Writing – review and editing. **Rachadol Thuephut**: Methodology, Investigation, Data curation, Formal analysis. **Kansuda Ketkrathok**: Investigation, Data curation, Formal analysis. **Chichaya Leerahakanch**: Investigation, Data curation, Formal analysis. **Patipan Chanwises**: Investigation, Data curation, Formal analysis. **Paiboon Jitprasertwong**: Methodology, Writing – review and editing. **Masahiro Yamada**: Methodology, Writing – review and editing. **Hiroshi Egusa**: Methodology, Writing – review and editing. **Phoonsuk Limraksasin**: Supervision, Methodology, Writing – review and editing.

## Data availability

The data underlying this article will be shared on reasonable request to the corresponding author.

## Funding

This work was supported by the (i) Suranaree University of Technology (SUT), (ii) Thailand Science Research and Innovation (TSRI) and (iii) National Science, Research and Innovation Fund (NSRF; Grant number: 215737, W.T.)

## Conflict of interests

None disclosed.

## Appendix Supplementary materials

Image, application 1

## Footnotes

Supplementary material associated with this article can be found in the online version at doi:10.1016/j.identj.2026.109581.
