# Supplementary material for: Matrix Stiffness Drives Aggressive Phenotype in Tongue Squamous Cell Carcinoma via Mechanotransduction–Stromal Signalling
Source: Int Dent J. 2026 Apr 25;76(4):109581. doi: 10.1016/j.identj.2026.109581 (PMC13134031; doi:10.1016/j.identj.2026.109581)
Supplement: Supplementary file 1 [file mmc1.docx]

**Supplementary Table 1.** Primer sequences used in this study

| Gene name | Primer sequence 5’-3’ | Product size | Accession number |
| --- | --- | --- | --- |
| *GAPDH* | (F) CAC TGC CAA CGT GTC AGT GGT G | 121 | NM_001289746.2 |
|  | (R) GTA GCC CAG GAT GCC CTT GAG |  |  |
| *E-cadherin (CDH1)* | (F) GCC GAG AGC TAC ACG TTC A | 88 | NM_001317185.2 |
|  | (R) GAC CGG TGC AAT CTT CAA A |  |  |
| *N-cadherin (CDH2)* | (F) CTC CAT GTG CCG GAT AGC | 92 | NM_001792.5 |
|  | (R) CGA TTT CAC CAG AAG CCT CTA C |  |  |
| *Vimentin (VIM)* | (F) GAG AAC TTT GCC GTT GAA GC | 163 | NM_003380.5 |
|  | (R) GCT TCC TGT AGG TGG CAA TC |  |  |
| *MMP2* | (F) TTG ACG GTA AGG ACG GAC TC | 153 | NM_001302509.2 |
|  | (R) ACT TGC AGT ACT CCC CAT CG |  |  |
| *MMP9* | (F) CAG TCC ACC CTT GTG CTC TT | 120 | NM_004994.3 |
|  | (R) CGA CTC TCC ACG CAT CTC TG |  |  |
| *Ki67* | (F) GAA AGA GTG GCA ACC TGC CTT C | 151 | NM_002417.5 |
|  | (R) GCA CCA AGT TTT ACT ACA TCT GCC |  |  |
| *YAP1* | (F) GCA CCT CTG TGT TTT AAG GGT CT | 195 | NM_006106.5 |
|  | (R) CAA CTT TTG CCC TCC TCC AA |  |  |
| *ITGA1* | (F) TGG TGG TGC TGC CCT CTT CTG | 218 | NM_181501.2 |
|  | (R) GTG AAT CTA GGG TGA CAC GGT AC |  |  |
| *ITGA5* | (F) TGC CTC CCT CAC CAT CTT C | 171 | NM_002205.5 |
|  | (R) TGC TTC TGC CAG TCC AGC |  |  |

GAPDH; Glyceraldehyde-3-phosphate dehydrogenase, MMP2; Matrix metalloproteinase-2, MMP9; Matrix metalloproteinase-9, YAP1; Yes-associated protein 1, ITGA1; Integrin alpha-1, ITGA5; Integrin alpha-5


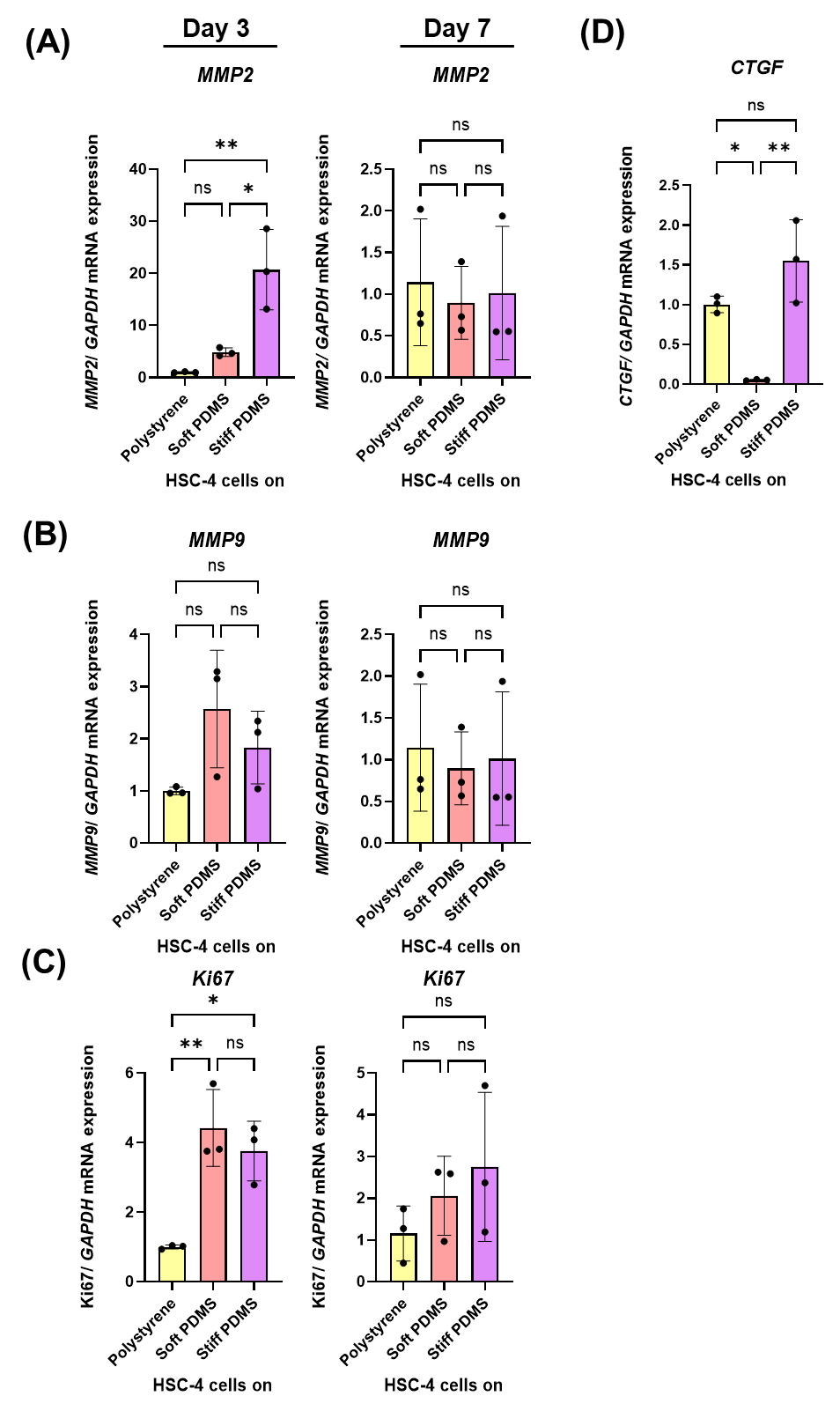


**Supplementary Figure 1. Substrate stiffness modulates EMT-related expression in HSC-4 cells.** HSC-4 cells were incubated under various stiffness conditions for 3 and 7 days. Real-time qRT-PCR was performed to detect mRNA expression levels of (A) *MMP2* (B) *MM9,* (C) *Ki67,* and (D) *CTGF*. The expression of *GAPDH* was used as an internal control. Data were statistically analyzed by one-way ANOVA followed by Tukey’s multiple comparison tests (n = 3: * *P < 0.05, * P < 0.01, *** P < 0.001, **** P < 0.0001, ns,* no significant difference). Data are presented as the mean ± standard deviation (SD). (D) Immunofluorescence analysis was performed to detect the vimentin protein expression (green). The nuclei (blue) were stained using DAPI, respectively. Scale bars: 20 μm. *MMP2*; Matrix metalloproteinase-2, *MMP9*; Matrix metalloproteinase-9, *CTGF*; connective tissue growth factor, *GAPDH*; glyceraldehyde-3-phosphate dehydrogenase, PDMS; polydimethylsiloxane.


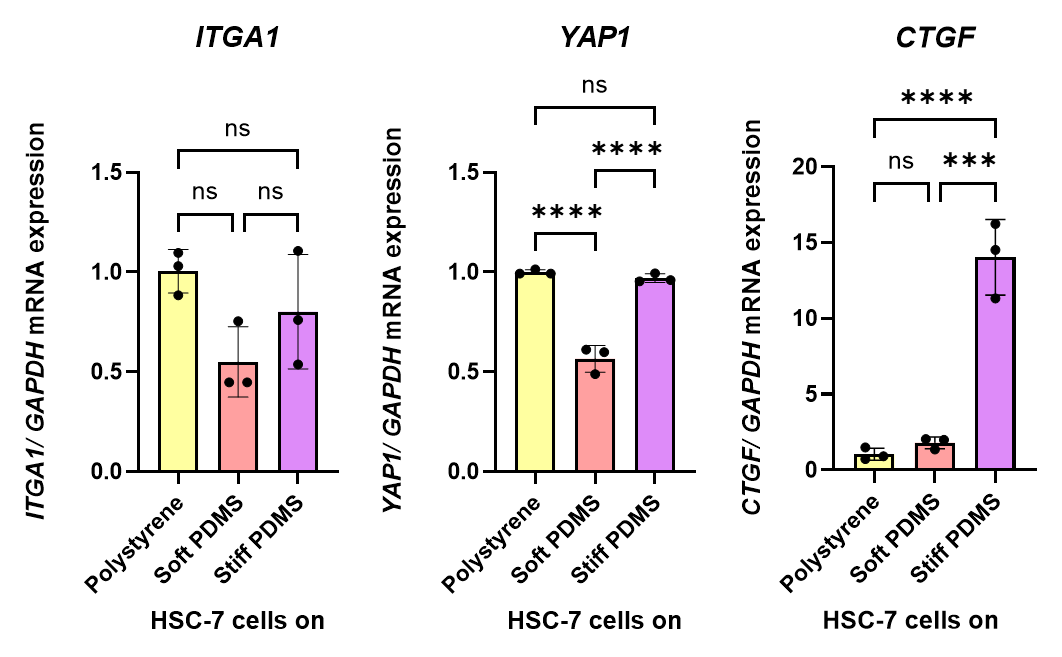


**Supplementary Figure 2. Mechanotransduction mechanism involved in the influence of substrate stiffness in HSC-7 cells.** Real-time RT-PCR was performed to detect mRNA expression levels of *YAP,* *ITGA1,* and *CTGF*. The expression of GAPDH was used as an internal control. Data were statistically analyzed by one-way ANOVA followed by Tukey’s multiple comparison tests (n = 3: * P < 0.05, * P < 0.01, *** P < 0.001, **** P < 0.0001, *ns*, no significant difference). Data are presented as the mean ± standard deviation (SD). *YAP1*; Yes-associated protein 1, *ITGA1*; Integrin alpha-1, *CTGF*; connective tissue growth factor, *GAPDH*; Glyceraldehyde-3-phosphate dehydrogenase, PDMS; polydimethylsiloxane.


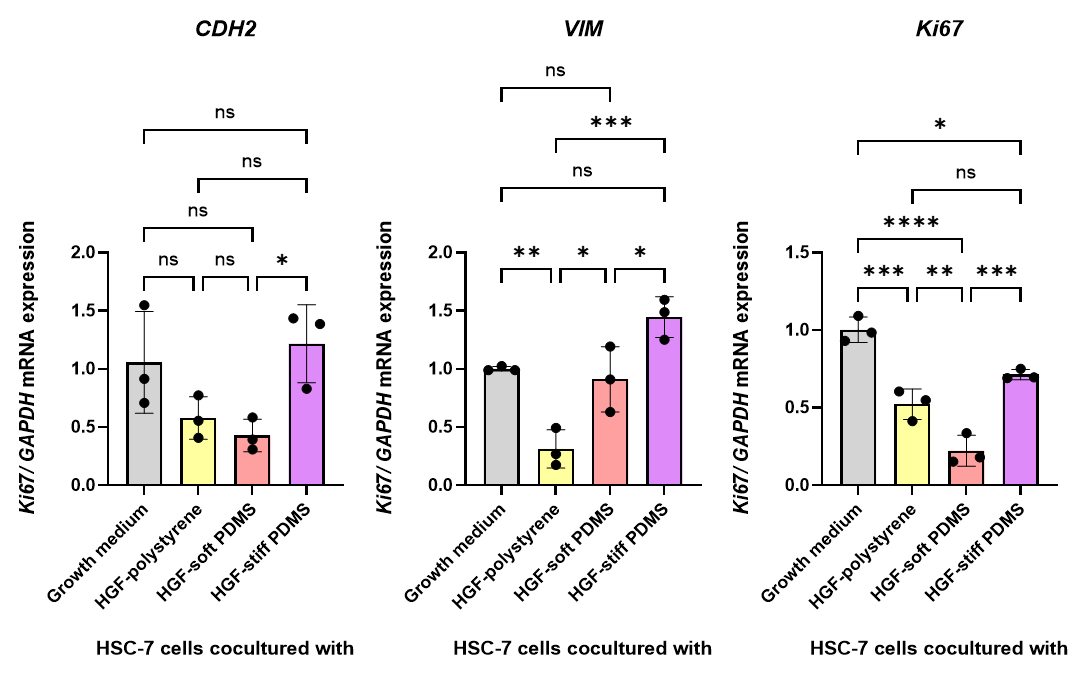


**Supplementary Figure 3. The effect of conditioned media of human gingival fibroblasts (HGF-CM) on the behavior of HSC-4 under different substrate stiffness.** Real-time RT-PCR was performed to detect mRNA expression levels of CDH2, VIM, and Ki67. The expression of GAPDH was used as an internal control. Data were statistically analyzed by one-way ANOVA followed by Tukey’s multiple comparison tests (n = 3: * P < 0.05, * P < 0.01, *** P < 0.001, **** P < 0.0001, ns, no significant difference). Data are presented as the mean ± standard deviation (SD). CDH2; N-cadherin, VIM; Vimentin, GAPDH; glyceraldehyde-3-phosphate dehydrogenase, PDMS; polydimethylsiloxane.
